# Association between asymptomatic infections and linear growth in 18–24‐month‐old Malawian children

**DOI:** 10.1111/mcn.13417

**Published:** 2022-09-16

**Authors:** Juho Luoma, Laura Adubra, Per Ashorn, Ulla Ashorn, Jaden Bendabenda, Kathryn G. Dewey, Lotta Hallamaa, Ryan Coghlan, William A. Horton, Heikki Hyöty, Emma Kortekangas, Kirsi‐Maarit Lehto, Kenneth Maleta, Andrew Matchado, Minyanga Nkhoma, Sami Oikarinen, Seppo Parkkila, Sami Purmonen, Yue‐Mei Fan

**Affiliations:** ^1^ Center for Child, Adolescent and Maternal Health Research, Faculty of Medicine and Health Technology Tampere University Tampere Finland; ^2^ Department of Paediatrics Tampere University Hospital Tampere Finland; ^3^ School of Public Health and Family Medicine, College of Medicine University of Malawi Blantyre Malawi; ^4^ Department of Nutrition, Institute for Global Nutrition University of California Davis California USA; ^5^ Research Center, Shriners Hospitals for Children Portland Oregon USA; ^6^ Department of Molecular and Medical Genetics Oregon Health and Science University Portland Oregon USA; ^7^ Department of Virology, Faculty of Medicine and Health Technology Tampere University Tampere Finland; ^8^ Fimlab Ltd. Tampere University Hospital Tampere Finland; ^9^ Clinical Medicine, Faculty of Medicine and Health Technology Tampere University Tampere Finland; ^10^ Present address: School of Global and Public Health Kamuzu University of Health Sciences Blantyre Malawi

**Keywords:** asymptomatic infection, childhood growth faltering, insulin‐like growth factor 1, structural equation modelling, stunting, systemic inflammation

## Abstract

Inadequate diet and frequent symptomatic infections are considered major causes of growth stunting in low‐income countries, but interventions targeting these risk factors have achieved limited success. Asymptomatic infections can restrict growth, but little is known about their role in global stunting prevalence. We investigated factors related to length‐for‐age *Z*‐score (LAZ) at 24 months by constructing an interconnected network of various infections, biomarkers of inflammation (as assessed by alpha‐1‐acid glycoprotein [AGP]), and growth (insulin‐like growth factor 1 [IGF‐1] and collagen X biomarker [CXM]) at 18 months, as well as other children, maternal, and household level factors. Among 604 children, there was a continuous decline in mean LAZ and increased mean length deficit from birth to 24 months. At 18 months of age, the percentage of asymptomatic children who carried each pathogen was: 84.5% enterovirus, 15.5% parechovirus, 7.7% norovirus, 4.6% rhinovirus, 0.6% rotavirus, 69.6% *Campylobacter*, 53.8% *Giardia lamblia*, 11.9% malaria parasites, 10.2% *Shigella*, and 2.7% *Cryptosporidium*. The mean plasma IGF‐1 concentration was 12.5 ng/ml and 68% of the children had systemic inflammation (plasma AGP concentration >1 g/L). *Shigella* infection was associated with lower LAZ at 24 months through both direct and indirect pathways, whereas enterovirus, norovirus, *Campylobacter*, *Cryptosporidium*, and malaria infections were associated with lower LAZ at 24 months indirectly, predominantly through increased systemic inflammation and reduced plasma IGF‐1 and CXM concentration at 18 months.

## Key messages

- Asymptomatic infections are common in apparently healthy children in rural Malawi.
- Asymptomatic infections are associated with reduced linear growth, mainly through systemic inflammation and reduced plasma concentration of insulin‐like growth factor 1 hormone.
- For achieving healthy growth at the population level, adequate nutrition programs may need to be complemented with comprehensive infection prevention and management.

## Graphical abstract

Inadequate diet and frequent symptomatic infections are considered major causes of growth stunting in low‐income countries, but interventions targeting these risk factors have achieved limited success. Asymptomatic infections are common in apparently healthy children in rural Malawi and are associated with reduced linear growth, mainly through systemic inflammation and reduced plasma concentration of insulin‐like growth factor 1 hormone.

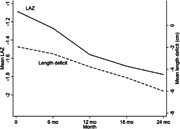

## 1 INTRODUCTION

Stunting, that is, faltering of linear growth, is common among children in low‐income countries. According to a recent estimate, 144 million or almost one‐fourth of all children <5 years of age in the world were stunted (United Nations Children's Fund, World Health Organization, & The World Bank, 2020). This condition is associated with an increased risk of mortality, morbidity, suboptimal development, adult‐life diseases, and loss of economic productivity (de Onis & Branca, 2016; Hoddinott et al., 2013). Given these adversities, the prevention of stunting has been set as a major global health priority (World Health Organization, 2012). With few exceptions, however, progress has been slower than desired (Development Initiatives, 2020).

One of the reasons for the slow progress may be the lack of information on the biological mechanisms that lead to childhood growth restriction in low‐income settings. Growth faltering often begins in utero and continues for the first 2 years of life. During these “first 1000 days,” inadequate feeding practices and factors related to infections are considered major risk factors (Stewart et al., 2013). Feeding problems could limit tissue accretion through reduced nutrient availability and infections could aggravate nutrient deficiency by inducing anorexia and reducing intestinal nutrient absorption (Dewey & Mayers, 2011). However, problems in nutrient intake and absorption may not adequately account for growth faltering in low‐income settings, as evidenced by the limited impact of dietary (Dewey & Adu‐Afarwuah, 2008) or water, sanitation, and hygiene interventions targeting intestinal infections (Pickering, 2019).

In addition to nutrient availability, linear growth is determined by biological pathways that regulate bone elongation and other tissue accretion. Hormones, such as insulin, thyroxin, growth hormone, insulin‐like growth factor 1 (IGF‐1), and sex steroids play a major role, with their relative importance changing over the course of childhood (Rosenbloom, 2007). After the first year of life and until puberty, IGF‐1 is believed to be the major hormonal regulator of human growth (Rosenbloom, 2007). Hence at this age, exposures that reduce children's plasma or tissue concentration of IGF‐1 are likely to reduce their length gain (Hawkes & Grimberg, 2015; Wong et al., 2016). One such exposure is systemic inflammation, which can block IGF‐1 synthesis in the liver (Walters & Griffiths, 2009). Inflammation is often caused by infections, and both symptomatic and asymptomatic infections have been associated with reduced plasma concentrations of IGF‐1 among infants and young children in low‐income settings (DeBoer et al., 2017; Maleta et al., 2020; Prendergast et al., 2014; Syed et al., 2018).

In an earlier publication, it has been reported that mean plasma IGF‐1 concentration was markedly lower and mean inflammatory biomarker concentration higher among 18‐ and 30‐month‐old children in Malawi than among same‐age children in a high‐income country (Maleta et al., 2020). Infection‐induced inflammation and reduction in plasma or tissue IGF‐1 concentration could thus be an important mechanism leading to growth faltering in low‐income settings, especially between 6 and 24 months of age, when both symptomatic and asymptomatic infections are common (Espo et al., 2002; Maleta et al., 2020; Platts‐Mills et al., 2015; Rogawski et al., 2018). In the current study, we aimed to characterise the frequency of asymptomatic bacterial, viral, and parasitic infections and their association with linear growth, among 18–24‐month‐old apparently healthy children in rural Malawi.

## 2 METHODS

### 2.1 Study design and concept map

This was a secondary analysis of data and biological samples that were prospectively collected as part of a dietary intervention trial in Malawi (iLiNS‐DYAD‐M) in which children in the intervention group received small‐quantity lipid‐based nutrient supplements starting at 6 months of age. The intervention stopped at 18 months and the children were followed up at 24 and 30 months of age. The details of the iLiNS‐DYAD‐M trial can be found elsewhere (ClinicalTrials.gov Identifier NCT01239693) (Ashorn, Alho, Ashorn, Cheung, Dewey, Gondwe, et al., 2015; Ashorn, Alho, Ashorn, Cheung, Dewey, Harjunmaa, et al., 2015).

In the current study, we analysed the association between biomarkers of infection, inflammation, and growth detected at 18 months of age and length‐for‐age Z‐score (LAZ) at 24 months of age. We chose this age interval as it is a period when infections are common (Fan et al., 2019; MAL‐ED Network Investigators, 2017; Platts‐Mills et al., 2015) and growth faltering still continues (Espo et al., 2002; Maleta et al., 2020; Victora et al., 2010) among children in low‐income countries.

Our conceptual model (Supporting Information: Figure 1) was based on widely adopted frameworks that include contextual factors, intermediate variables, and proximal determinants of the child's nutritional status (Black et al., 2008; Buyuk et al., 2021; Stewart et al., 2013). We included household assets and parental education as factors reflecting the environmental and socioeconomic context. We hypothesised that suboptimal living conditions would increase exposure to infections and related factors. We further assumed that intestinal infections would predict lower LAZ in the subsequent 6 months through increased intestinal inflammation (as assessed by fecal calprotectin and alpha‐1 antitrypsin [A1AT]), whereas malaria and invasive bacterial infections would induce a systemic inflammatory response (assessed by plasma alpha‐1‐acid glycoprotein [AGP]), which, in turn, would negatively affect plasma IGF‐1 and collagen X biomarker (CXM) levels. Systemic inflammation was indicated when the AGP concentration was >1 g/L (Thurnham et al., 2010). The model also included three earlier identified predictors of attained LAZ in childhood, that is, the child's LAZ and weight‐for‐length *Z*‐score (WLZ) at the beginning of the growth follow‐up period (18 months), and maternal height.

To characterise growth, both LAZ and the difference in length in centimetres from the reference median (called length deficit in this study) were calculated. LAZ was calculated using the WHO 2006 Child Growth Standards (World Health Organization, 2006). Using the same references, length deficit was calculated as the difference between the measured length and the median age‐ and sex‐specific length obtained from the reference data. The use of absolute height differences has been suggested as an alternative indicator of linear growth retardation, as it may be more appropriate in reflecting the potential accumulation of growth deficit over time (Leroy et al., 2015; Lundeen et al., 2014).

### 2.2 Sample and data collection

#### 2.2.1 Biological sample collection

Biological sample collection was conducted at 18 months of age. All samples were collected from children who were apparently healthy at the time of biospecimen collection. Nurses in the clinic collected nonfasting blood samples from the children's antecubital vein and a laboratory technician separated plasma into storage vials. Mothers collected stool samples from their children at home. If a child had diarrhoea, no stool sample was collected, and the visit was postponed by 2 weeks. The research assistants picked up collected stool samples on the same day and placed them in cooler bags. The laboratory technician aliquoted them to cryovial tubes for storage and frozen at −20°C. Within 48 h, samples were transported to a central laboratory where they were frozen at −80°C and stored. Plasma and stool samples were later shipped on dry ice for analysis to Tampere University, Finland and the University of California, Davis, US.

#### 2.2.2 Laboratory analyses

Stool samples were assayed at Tampere University using commercially available ELISA kits for calprotectin (HycultBiotech) and A1AT (PromoCell GmbH), which are markers of intestinal inflammation and permeability, respectively (Kosek et al., 2013). *Campylobacter* (de Boer et al., 2015), *Shigella* (Vu et al., 2004), *Cryptosporidium spp.*, *Giardia* (Nurminen et al., 2015), enterovirus, rhinovirus, parechovirus, norovirus, and rotavirus (Krogvold et al., 2015) infections were detected using an in‐house real‐time polymerase chain reaction assay. These pathogens are widely spread and reflect hygienic conditions where the children are living (Fan et al., 2019). Malaria was diagnosed on‐site from finger‐prick blood samples using the rapid diagnostic test Clearview Malaria Combo (British Biocell International Ltd.).

Carrier‐protein‐free IGF‐1 concentration was analysed at Tampere University from stored plasma samples using commercial MILLIPLEX®MAP HIGF‐I, II Magnetic Bead Panel Kit (Cat. # HIGFMAG‐52K; EMD Millipore Corporation), according to the manufacturer's instructions.

Plasma CXM concentrations were analysed with in‐house ELISA kits at Shriners Hospital for Children from plasma samples. AGP was analysed on a Roche Cobas 6000 analyzer (Roche Diagnostics) at the University of California, Davis.

#### 2.2.3 Environmental variables

A household wealth proxy was created by principal component analysis using sociodemographic information that was collected through personal interviews with mothers. Information used for the principal component were building materials of the house, water source of the household, sanitation, electricity, and cooking fuel. Unimproved sanitation was classified as having a regular pit latrine or no latrine in the household. The unimproved water source was classified as having a water source from a lake, pond, or unprotected well. The education of the mother was expressed as completed years at school. Maternal height was measured at enrolment. We did not include exclusivity of breastfeeding during the first 6 months as a covariate because there were a lot of missing datapoints for this variable, which would have significantly reduced the effective sample size. The rate of breastfeeding is high in Malawi, and as in our study 100% of the mothers reported still breastfeeding at 6 months and 92% at 18 months; we decided not to include breastfeeding in general due to the lack of variability.

### 2.3 Statistical analyses

To investigate the relationship between a child's LAZ at 24 months of age and immediate continuous predictor variables, we ranked our study participants based on their predictor variable value and grouped them in five quintiles by this rank. This was done separately for each predictor variable. We then calculated the mean LAZ at 24 months of age in the respective quintiles and tested a hypothesis about an increasing or decreasing trend with an extended Wilcoxon rank sum test (Cuzick, 1985).

We investigated the distribution shapes of the variables before linear modelling. To satisfy the assumption of normality for linear modelling, skewed continuous variables were transformed using natural logarithm transformation.

To investigate the association between a child's LAZ at 24 months of age and underlying dichotomous predictor variables – various viral, bacterial, and parasitic infections, we compared means and standard deviations between groups and tested the hypothesis of difference in means between groups by using Student's *t*‐test.

After the initial bivariate relation investigation, we fitted single variable ordinary least‐squares regression models to estimate an effect size between every single predictor and the response variable for each of the intermediate outcome variables and for the main outcome variable, LAZ at 24 months of age. We examined the proportion and patterns of missing data and used multiple imputations with chained equations for missing data.

We selected variables for the structural equation modelling (SEM), by constructing a full model based on the concept map and then removing variables by the backward selection, using the Akaike information criterion to compare the models (Akaike, 1973). Variables were standardised before model fitting. To assess model fit, we estimated the comparative fit index (CFI), the Tucker–Lewis index (TLI) and the root mean square error of approximation (RMSEA). Values above 0.90 for CFI and TLI indicate a good fit, and a value below 0.05 for RMSEA was considered a good fit. Finally, we constructed a model visualisation using results from the SEM: coefficients that were considered statistically significant (*p* < 0.05) were included in the graph.

All statistical analyses were done with Stata version 15.1 (StataCorp).

## 3 RESULTS

Of the 790 live‐born Malawian infants, a total of 604 (77%) children were included in the current study; reasons for exclusion were death (49), dropout (55), and missed visits at 24 months (82) (Supporting Information: Figure 2). A total of 48.2% of the study participants were boys and 91.5% lived in households with unimproved sanitation facilities. Mean maternal height was 156.1 cm, and 12.5% of the mothers were HIV positive. The excluded children had similar baseline characteristics compared to the children included in the analysis, except for a greater proportion of primiparous mothers (36.0% vs. 17.8%, *p* < 0.001) and higher mean educational achievement among mothers (4.8 years vs. 3.6 completed years in school, *p* < 0.001) (Supporting Information: Table 1). For most variables, less than 5% of values were missing, for CXM, this proportion was 15.1% (Supporting Information: Table 2).

**Figure 1:**
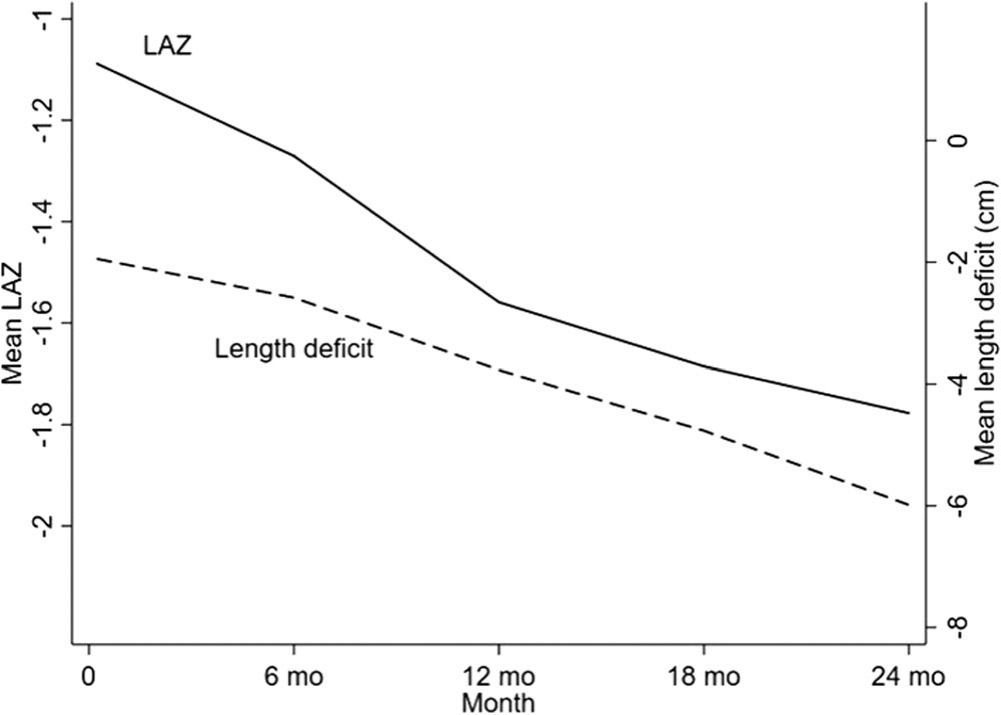
Mean LAZ and length deficit (cm) relative to the WHO standard (1–23 months) by completed month (*n* = 604). The solid line indicates participants' mean LAZ. The dashed line indicates the absolute difference between the mean length in the study sample and the 50th centile in the reference population (called length deficit here). LAZ, length‐for‐age *Z*‐score; WHO, World Health Organisation.

The participants' mean LAZ was −1.1 at 2 weeks of age, −1.7 at 18 months, and −1.8 at 24 months. The absolute difference between the mean length in the study sample and the 50th centile in the reference population (called length deficit in this publication) was 2.0 cm at two weeks and 6.0 cm at 2 years of age (Figure 1).

### 3.1 Predictor variables and their bivariate associations with LAZ at 24 months

At 18 months of age, the children's mean plasma concentration was 12.5 ng/ml for IGF‐1, 28.3 ng/ml for CXM, and 1.4 g/L for AGP. Sixty‐eight percent of the children had elevated plasma AGP (>1 g/L). Children's plasma concentrations of IGF‐1 and CXM were positively and plasma AGP concentrations were inversely associated with their LAZ at 24 months (*p* < 0.001). There was no association between the children's intestinal inflammation markers at 18 months of age and their LAZ at 24 months of age (Table 1).

**Table 1:** The association between selected biomarkers detected at 18 months and children's LAZ at 24 months

Mean LAZ at 24 months, by the participants' rank in the indicated predictor variable value distribution at 18 months							
Predictor variable	Mean ± SD concentration	Lowest quintile^a^	Second quintile	Third quintile	Fourth quintile	Highest quintile	*p*‐Value*
Plasma IGF‐1	12.5 ± 7.6 ng/ml	−2.13	−1.96	−1.70	−1.70	−1.38	<0.001
Plasma CXM	28.3 ± 9.1 ng/ml	−2.17	−1.83	−1.59	−1.66	−1.59	<0.001
Plasma AGP	1.4 ± 0.6 g/L	−1.59	−1.61	−1.63	−1.89	−2.12	<0.001
Fecal calprotectin	227 ± 326 µg/g	−1.74	−1.63	−1.71	−1.84	−1.92	0.09
Fecal A1AT	7.4 ± 16.2 mg/dl	−1.74	−1.89	−1.87	−1.68	−1.64	0.22

Abbreviations: A1AT, alpha‐1 antitrypsin; AGP, alpha‐1‐acid glycoprotein; CXM, collagen X biomarker; IGF‐1, insulin‐like growth factor; LAZ, length‐for‐age *Z*‐score.

^a^ Children within the lowest quintile of the inflammation marker (lowest 20% of values).

* *p*‐value obtained using Cuzick's Wilcoxon‐type test for trend.

At 18 months of age, 84.5% of the children had a positive stool test result for enterovirus, 15.5% for parechovirus, 7.7% for norovirus, 4.6% for rhinovirus, and 0.6% for rotavirus. There was no association between the detection of any of these viruses in children's stool at 18 months and their LAZ at 24 months of age (Table 2).

**Table 2:** The association between viral, bacterial, and parasitic infections detected at 18 months and children's LAZ at 24 months

Microbe	The proportion of positive samples	Children with negative test results (number of samples)	Children with positive test results (number of samples)	Difference (95% CI)	*p‐*Value*
Mean ± SD LAZ at 24 months					
Enterovirus	84.5%	−1.82 ± 1.26 (91)	−1.76 ± 1.01 (495)	−0.07 (−0.30 to 0.17)	0.59
Parechovirus	15.5%	−1.74 ± 1.07 (495)	−1.92 ± 0.94 (91)	0.18 (−0.06 to 0.42)	0.13
Norovirus	7.7%	−1.78 ± 1.05 (541)	−1.62 ± 1.04 (45)	−0.15 (−0.47 to 0.17)	0.35
Rhinovirus	4.6%	−1.75 ± 1.04 (559)	−2.05 ± 1.26 (27)	0.29 (−0.12 to 0.70)	0.24**
Rotavirus	0.6%	−1.77 ± 1.05 (582)	−1.89 ± 1.63 (4)	0.12 (−2.47 to 2.70)	0.86**
Bacterial species					
*Shigella*	10.2%	−1.73 ± 1.03 (527)	−2.11 ± 1.11 (60)	0.39 (0.11–0.67)	0.006
*Campylobacter*	69.6%	−1.65 ± 1.09 (179)	−1.82 ± 1.02 (410)	0.17 (−0.01 to 0.36)	0.07
Parasitic species					
*Cryptosporidium*	2.7%	−1.77 ± 1.05 (570)	−1.80 ± 1.11 (16)	0.03 (−0.50 to 0.55)	0.85**
*Giardia lamblia*	53.8%	−1.78 ± 1.18 (271)	−1.76 ± 0.93 (315)	−0.02 (−0.20 to 0.15)	0.81
Blood malaria parasitemia	11.9%	−1.75 ± 1.04 (511)	−1.88 ± 0.92 (69)	0.13 (−0.10 to 0.37)	0.27

Abbreviations: CI, confidence interval; LAZ, length‐for‐age *Z*‐score.

* *p*‐value obtained using Student's *t*‐test unless otherwise specified.

** *p*‐value obtained using Wilcoxon sum of ranks test.

At 18 months of age, 10.2% of the children had a positive stool test result for *Shigella*, 69.6% for *Campylobacter*, 2.7% for *Cryptosporidium*, and 53.8% for *Giardia lamblia*. The prevalence of malaria parasitemia was 11.9%. Children who had *Shigella* species detected in their stools at 18 months of age had on average (95% CI) 0.39 (0.11 to 0.67, *p* = 0.006) lower LAZ at 24 months of age than children whose stools gave a negative test result for *Shigella*. There was no association between the detection of any of other above bacteria and parasites in children's stools or malaria parasitemia in their blood and their LAZ at 24 months of age (Table 2).

Children's LAZ at 24 months of age was strongly associated with their LAZ and WLZ at 18 months, maternal height, and family wealth (*p* < 0.001). In contrast, there was no association between maternal education and child LAZ at 24 months of age (Supporting Information: Table 3).

### 3.2 Pathway model for determinants of LAZ at 24 months

**Figure 2:**
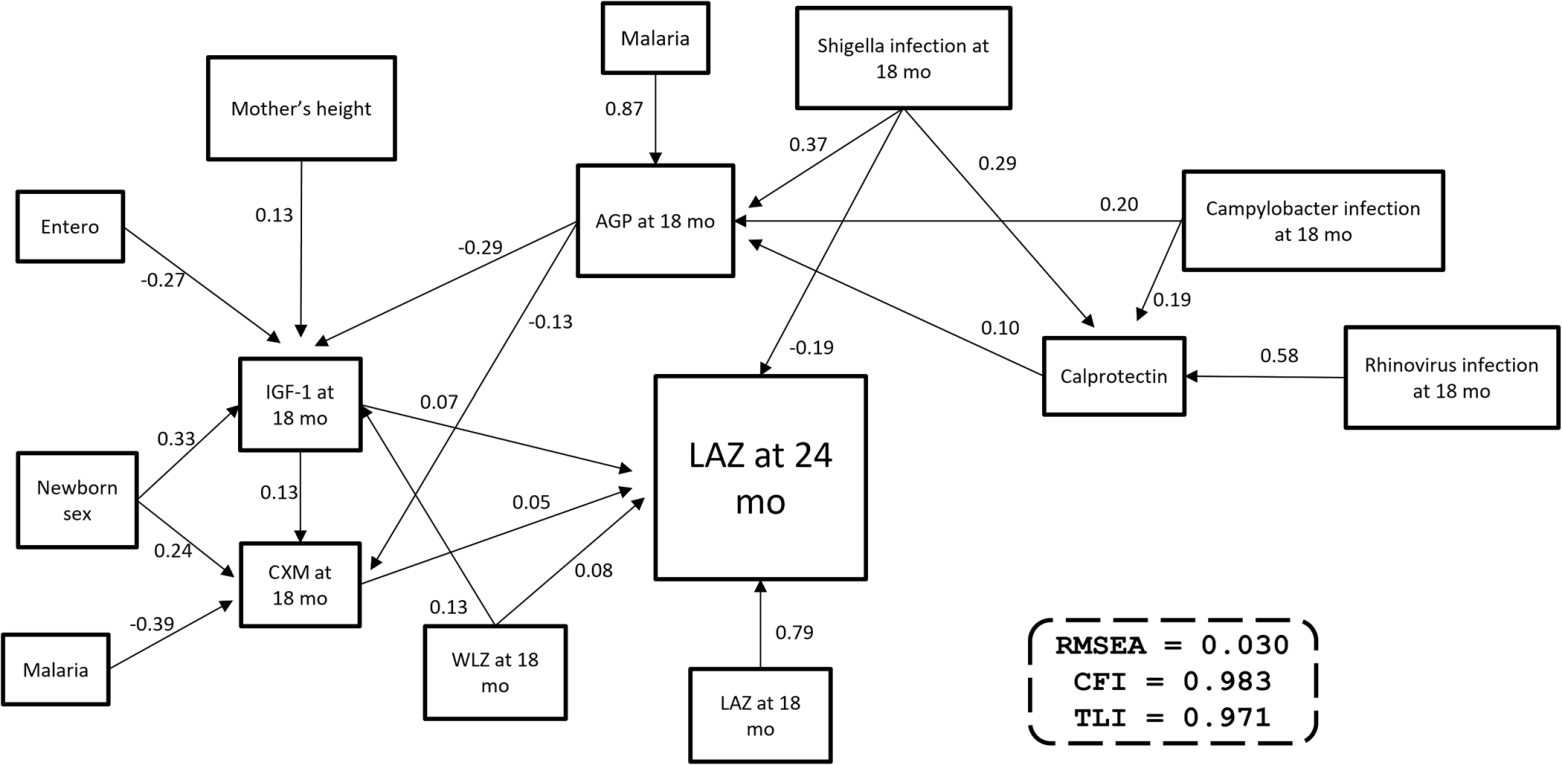
Pathway model for the direct and indirect associations with LAZ at 24 months of age (*n* = 604). The model was done with structural equation modelling. The numbers next to the lines stand for the model coefficients. The model used standardised variables, and the negative associations are indicated with a minus sign before the number. AGP, alpha‐1‐acid glycoprotein; CFI, comparative fit index; IGF‐1, insulin‐like growth factor 1; LAZ, length‐for‐age *Z*‐score; RMSEA, root mean square error of approximation; TLI, Tucker–Lewis index; WLZ, weight‐for‐length *Z*‐score.

In a pathway model, LAZ at 24 months of age was directly predicted by the children's LAZ and WLZ at 18 months, child plasma IGF‐1 and CXM concentration at 18 months, and presence of intestinal *Shigella* infection at 18 months (Figure 2, full details of SEM in Supporting Information: Table 4). Plasma IGF‐1 and CXM concentrations were positively associated with maternal height, female sex, and WLZ at 18 months, and inversely associated with the children's concomitant plasma concentration of AGP and enterovirus. Malaria, *Shigella*, *Campylobacter*, and fecal calprotectin concentration at 18 months of age were inversely associated with the children's LAZ at 24 months, through their association with plasma AGP concentration (Figure 2). Unlike in our conceptual model, the final, reported model did not include any environmental or socioeconomic variables. In sensitivity analysis, asset index and parental education added as covariates in the model had no effect on model fit, effects, or statistical significances (data not shown).

## 4 DISCUSSION

The current study investigated associations between asymptomatic infections and linear growth in rural Malawi. In a sample of 604 children, early childhood growth was characterised by a steady decline in LAZ from birth to 24 months. At 18 months of age, there was a high prevalence of asymptomatic viral, bacterial, and parasitic infections. *Shigella* infection was associated with lower LAZ at 24 months through both direct and indirect pathways, whereas enterovirus, norovirus, *Campylobacter*, *Cryptosporidium*, and malaria infections were associated with lower LAZ at 24 months indirectly, predominantly through increased systemic inflammation and reduced plasma IGF‐1 and CXM concentration at 18 months. The child's earlier LAZ and WLZ, as well as plasma concentration of CXM were other independent predictors of LAZ at 24 months.

Internal validity could have been compromised by missing data, or the employed analytical method. External validity could have been affected by the choice of variables in the path analyses. However, children excluded from the analysis and those with missing data had on average similar baseline characteristics compared to participants who provided data, data missingness was rare and random, and we used multiple imputations to model the missing values (Buuren & Groothuis‐Oudshoorn, 2011). The choice of variables was based on previous studies (Dewey et al., 2005; Maleta et al., 2020; Ozaltin et al., 2010), and the use of the SEM technique allowed simultaneous testing of several relationships (DiLalla, 2008). The cross‐sectional nature of our study is a limitation that does not allow for conclusions on causality. However, the prospective design helps to mitigate the risk of reverse causality by allowing associations in the path model to be drawn from variables measured at earlier time points (e.g., infections at 18 months) to variables measured at later time points (LAZ at 24 months). Finally, although we collected samples in apparently healthy children, part of whom received a dietary intervention, intestinal bacterial, viral, and parasitic infections were prevalent in our study, similar to results from another study we conducted in the same region (Fan et al., 2019). These pathogens are commonly spread and reflect the poor hygienic conditions in which the children live. Therefore, we believe that our results can be generalised to children living in similar resource‐constrained settings.

The association between symptomatic infections and growth faltering in low‐income settings is well documented (Brander et al., 2019; Checkley et al., 2008; Jackson & Black, 2017; Weisz et al., 2011). Less is known about asymptomatic infections, but available evidence suggests that they are common in populations with a high prevalence of stunting. Studies conducted in the Democratic Republic of Congo (Lufungulo Bahati et al., 2020) and Burkina Faso (Natama et al., 2018) reported an 18%–31% prevalence of asymptomatic malaria infection among 3–23‐month‐old children. In a Ghanaian study (Crookston et al., 2010), approximately one‐third of apparently healthy under 5‐year‐old children tested positive for malaria. Almost the same proportion of 12‐ and 18‐month‐old Malawian children with no disease symptoms had rhinovirus, parechovirus, norovirus, or *Giardia lamblia* in their stools and the prevalence of enterovirus was as high as 81% (Fan et al., 2019). Bacterial enteropathogens, such as *E. coli* (51% prevalence), *Giardia lamblia* (30%), *Campylobacter* (28%), and *Shigella* (11%), were frequently detected in the absence of diarrhoea among 2–24‐month‐old children at several MALED‐study sites in South Asia, Latin America and Sub‐Saharan Africa (Platts‐Mills et al., 2015). Similar to the results from the current study and an earlier finding for Malawian children with asymptomatic *Giardia lamblia* infection (Lehto et al., 2019), subclinical infections with these four pathogens were associated with substantial decrements in LAZ at 2 years (Rogawski et al., 2018). These results corroborate our findings and suggest that asymptomatic infections contribute markedly to childhood growth faltering in many low‐income settings.

Previous studies (Kosek et al., 2013) have suggested an association between fecal markers of gut function and LAZ at 24 months. We, however, did not observe such an association in our sample. The possible reasons for the differences between our study and other previous studies are children's health status (healthy/diarrhoea), different fecal markers used, background inflammation, and dietary factors.

Systemic inflammation, leading to reduced plasma and tissue concentration of IGF‐1 and possibly other growth‐promoting hormones, constitutes a possible mechanism linking infections to growth faltering. An earlier report (Maleta et al., 2020) documented an inverse association between infections, systemic inflammation, and plasma IGF‐1 concentration, and that seasonal changes in children's plasma IGF‐1 concentration coincided with changes in their length gain velocity. Similarly, systemic inflammation was more common among stunted than nonstunted 6–18‐months‐old Zimbabwean children (Prendergast et al., 2014). Inflammation was inversely associated with children's plasma IGF‐1 concentration also among 6–18‐months‐old Tanzania (Syed et al., 2018) and Zimbabwean (Prendergast et al., 2014) children. The findings are consistent with animal data suggesting that systemic inflammation blocks growth‐hormone stimulated IGF‐1 expression in the liver, leading to decreased hepatic IGF‐1 synthesis and ultimately restriction of growth (Ballinger et al., 2000; Wong et al., 2016).

The other variables identified as independent direct predictors of the child's LAZ at 24 months of age in our model were consistent with previous literature. Linear growth is known to be partly regulated by body mass or fatness (Dewey et al., 2005), and weight‐for‐length stimulates growth hormone and IGF‐I production in children (Benyi & Sävendahl, 2017). In our statistical model, we used LAZ at 18 months of age to standardise time points when the predictor values were collected but using birth LAZ gave essentially similar results (data not shown).

Probably the least studied direct predictor of attained LAZ in our model was the plasma concentration of CXM. This protein is considered a marker of chondrocyte growth and differentiation and its plasma concentration has been associated with bone growth velocity in children (Coghlan et al., 2017, 2021). Our results suggest that plasma CXM concentration has a direct positive association with linear growth, and it also serves as an intermediary outcome variable for inflammation and IGF‐1 concentration. However, further research is needed to clarify the role of CXM in the growth faltering pathway.

Our study focused on linear growth between 18 and 24 months of age. Whilst this is an age interval when growth faltering continues in many low‐income settings (Victora et al., 2010), the results cannot necessarily be extrapolated to earlier ages, during which a significant proportion of childhood growth faltering takes place. In the fetal period and early infancy, length gain is more associated with maternal nutrition whereas it becomes dependent on the child's own growth hormone production only in later infancy or early childhood (Karlberg, 1989; Rosenbloom, 2007). If their negative impact on length gain is mediated entirely by downregulation of growth‐hormone‐induced IGF‐1 expression, infections would not be expected to reduce growth velocity before the shift to the growth‐hormone‐dependent childhood growth phase. However, infections have also been associated with fetal growth restriction (Ashorn et al., 2018) and growth faltering between 6 and 18 months of age (Prendergast et al., 2014; Syed et al., 2018). While the relative contribution of infections to linear growth faltering may vary by the child's age, it thus seems likely that they have some impact also before 18 months.

In summary, asymptomatic infections were common in apparently healthy children in rural Malawi, and infection‐induced systemic inflammation was associated with reduced plasma IGF‐1 concentration, leading to impaired linear growth. In this and other low‐income areas where sanitary conditions are suboptimal and malaria may be endemic, adequate nutrition programs may need to be complemented with comprehensive infection prevention and management, to achieve healthy growth among the entire child population.

## AUTHOR CONTRIBUTIONS

Laura Adubra, Yue‐Mei Fan, and Per Ashorn conceptualised and designed the study, drafted the initial manuscript, and reviewed and revised the manuscript. Juho Luoma conceptualised the study, carried out the statistical analysis, drafted the initial manuscript, and reviewed and revised the manuscript. Ulla Ashorn and Kathryn G. Dewey conceptualised and designed the study, and reviewed and revised the manuscript critically. Jaden Bendabenda, Kenneth Maleta, Emma Kortekangas, Lotta Hallamaa, Kirsi‐Maarit Lehto, Andrew Matchado, and Minyanga Nkhoma contributed to the conception and design, coordinated and supervised data collection, and critically reviewed and revised the manuscript. Sami Purmonen, Seppo Parkkila, Sami Oikarinen, Heikki Hyöty, William A. Horton, and Ryan Coghlan reviewed and revised the manuscript critically. All authors reviewed and approved the final manuscript as submitted and agree to be accountable for all aspects of the work.

## CONFLICT OF INTEREST

William A. Horton is listed as an inventor on a patent application, “Type X collagen assay and methods of use thereof,” submitted by Shriners Hospitals for Children. He has consulted for and/or received speaker honoraria from BioMarin, TherAchon (now owned by Pfizer), Ascendis, QED, Relay Therapeutics, Fortress Biotech, OPKO, and Medicell. The other authors have no conflict of interest.

## ETHICS STATEMENT

The ethical approval was given by the College of Medicine Research Ethics Committee, University of Malawi and the Ethics Committee of Pirkanmaa Hospital District, Finland. Only participants whose caregivers gave informed consent were enroled in the study.

## Supporting information

Supporting information.

## Data Availability

Deidentified individual participant data will be made available upon publication at https://doi.org/10.5281/zenodo.4633329.

## References

[mcn13417-bib-0001] Akaike, H. (1973). Information theory and an extension of the maximum likelihood principle. In B. N. Petrov , & F. Csaki (Eds.), Proceedings of the 2nd International Symposium on Information Theory (pp. 267–281). Akademiai Kiado.

[mcn13417-bib-0002] Ashorn, P. , Alho, L. , Ashorn, U. , Cheung, Y. B. , Dewey, K. G. , Gondwe, A. , Harjunmaa, U. , Lartey, A. , Phiri, N. , Phiri, T. E. , Vosti, S. A. , Zeilani, M. , & Maleta, K. (2015). Supplementation of maternal diets during pregnancy and for 6 months postpartum and infant diets thereafter with Small‐quantity Lipid‐based nutrient supplements does not promote child growth by 18 months of age in rural Malawi: A randomized controlled trial‐4. The Journal of Nutrition, 145(6), 1345–1353. 10.3945/jn.114.207225 25926413

[mcn13417-bib-0003] Ashorn, P. , Alho, L. , Ashorn, U. , Cheung, Y. B. , Dewey, K. G. , Harjunmaa, U. , Lartey, A. , Nkhoma, M. , Phiri, N. , Phuka, J. , Vosti, S. A. , Zeilani, M. , & Maleta, K. (2015). The impact of lipid‐based nutrient supplement provision to pregnant women on newborn size in rural Malawi: A randomized controlled trial. The American Journal of Clinical Nutrition, 101(2), 387–397. 10.3945/ajcn.114.088617 25646337

[mcn13417-bib-0004] Ashorn, P. , Hallamaa, L. , Allen, L. H. , Ashorn, U. , Chandrasiri, U. , Deitchler, M. , Doyle, R. , Harjunmaa, U. , Jorgensen, J. M. , Kamiza, S. , Klein, N. , Maleta, K. , Nkhoma, M. , Oaks, B. M. , Poelman, B. , Rogerson, S. J. , Stewart, C. P. , Zeilani, M. , & Dewey, K. G. (2018). Co‐causation of reduced newborn size by maternal undernutrition, infections, and inflammation. Maternal & Child Nutrition, 14(3):e12585. 10.1111/mcn.12585 29316198 PMC6055652

[mcn13417-bib-0005] Ballinger, A. B. , Azooz, O. , El‐Haj, T. , Poole, S. , & Farthing, M. J. G. (2000). Growth failure occurs through a decrease in insulin‐like growth factor 1 which is independent of undernutrition in a rat model of colitis. Gut, 46(5), 695–700. 10.1136/gut.46.5.695 PMC1727919 10764714

[mcn13417-bib-0006] Benyi, E. , & Sävendahl, L. (2017). The physiology of childhood growth: Hormonal regulation. Hormone Research in Paediatrics, 88(1), 6–14. 10.1159/000471876 28437784

[mcn13417-bib-0007] Black, R. E. , Allen, L. H. , Bhutta, Z. A. , Caulfield, L. E. , de Onis, M. , Ezzati, M. , Mathers, C. , & Rivera, J. , Maternal and Child Undernutrition Study Group . (2008). Maternal and child undernutrition: global and regional exposures and health consequences. Lancet, 371(9608), 243–260. 10.1016/S0140-6736(07)61690-0 18207566

[mcn13417-bib-0008] Brander, R. L. , Pavlinac, P. B. , Walson, J. L. , John‐Stewart, G. C. , Weaver, M. R. , Faruque, A. S. G. , Zaidi, A. K. M. , Sur, D. , Sow, S. O. , Hossain, M. J. , Alonso, P. L. , Breiman, R. F. , Nasrin, D. , Nataro, J. P. , Levine, M. M. , & Kotloff, K. L. (2019). Determinants of linear growth faltering among children with moderate‐to‐severe diarrhea in the Global Enteric Multicenter Study. BMC Medicine, 17(1), 214. 10.1186/s12916-019-1441-3 31767012 PMC6878715

[mcn13417-bib-0009] Buuren, S. , & Groothuis‐Oudshoorn, C. (2011). MICE: Multivariate imputation by chained equations in R. Journal of Statistical Software, 45, 1–67. 10.18637/jss.v045.i03

[mcn13417-bib-0010] Buyuk, G. N. , Kansu‐Celik, H. , Kaplan, Z. A. O. , Kisa, B. , Ozel, S. , & Engin‐Ustun, Y. (2021). Risk factors for intrapartum cesarean section delivery in low‐risk multiparous women following at least a prior vaginal birth (Robson classification 3 and 4. Revista Brasileira de Ginecologia e Obstetrícias, 43(6), 436–441. 10.1055/s-0041-1731378 PMC10411140 34318468

[mcn13417-bib-0011] Checkley, W. , Buckley, G. , Gilman, R. H. , Assis, A. M. , Guerrant, R. L. , Morris, S. S. , Mølbak, K. , Valentiner‐Branth, P. , Lanata, C. F. , & Black, R. E. (2008). Multi‐country analysis of the effects of diarrhoea on childhood stunting. International Journal of Epidemiology, 37(4), 816–830. 10.1093/ije/dyn099 18567626 PMC2734063

[mcn13417-bib-0012] Coghlan, R. F. , Oberdorf, J. A. , Sienko, S. , Aiona, M. D. , Boston, B. A. , Connelly, K. J. , Bahney, C. , LaRouche, J. , Almubarak, S. M. , Coleman, D. T. , Girkontaite, I. , von der Mark, K. , Lunstrum, G. P. , & Horton, W. A. (2017). A degradation fragment of type X collagen is a real‐time marker for bone growth velocity. Science Translational Medicine, 9(419), eaan4669. 10.1126/scitranslmed.aan4669 29212713 PMC6516194

[mcn13417-bib-0013] Coghlan, R. F. , Olney, R. C. , Boston, B. A. , Coleman, D. T. , Johnstone, B. , & Horton, W. A. (2021). Norms for clinical use of CXM, a real‐time marker of height velocity. The Journal of Clinical Endocrinology & Metabolism, 106(1), e255–e264. 10.1210/clinem/dgaa721 33034649 PMC7765635

[mcn13417-bib-0014] Crookston, B. T. , Alder, S. C. , Boakye, I. , Merrill, R. M. , Amuasi, J. H. , Porucznik, C. A. , Stanford, J. B. , Dickerson, T. T. , Dearden, K. A. , Hale, D. C. , Sylverken, J. , Snow, B. S. , Osei‐Akoto, A. , & Ansong, D. (2010). Exploring the relationship between chronic undernutrition and asymptomatic malaria in Ghanaian children. Malaria Journal, 9(1), 39. 10.1186/1475-2875-9-39 20122258 PMC2837055

[mcn13417-bib-0015] Cuzick, J. (1985). A Wilcoxon‐type test for trend. Statistics in Medicine, 4(1), 87–90. 10.1002/sim.4780040112 3992076

[mcn13417-bib-0016] de Boer, P. , Rahaoui, H. , Leer, R. J. , Montijn, R. C. , & van der Vossen, J. M. B. M. (2015). Real‐time PCR detection of *Campylobacter* spp.: A comparison to classic culturing and enrichment. Food Microbiology, 51, 96–100. 10.1016/j.fm.2015.05.006 26187833

[mcn13417-bib-0017] DeBoer, M. D. , Scharf, R. J. , Leite, A. M. , Férrer, A. , Havt, A. , Pinkerton, R. , Lima, A. A. , & Guerrant, R. L. (2017). Systemic inflammation, growth factors, and linear growth in the setting of infection and malnutrition. Nutrition, 33, 248–253. 10.1016/j.nut.2016.06.013 27712965 PMC5193489

[mcn13417-bib-0018] de Onis, M. , & Branca, F. (2016). Childhood stunting: A global perspective. Maternal & Child Nutrition, 12(Suppl 1), 12–26. 10.1111/mcn.12231 27187907 PMC5084763

[mcn13417-bib-0019] Development Initiatives . (2020). *Global nutrition report: Action on equity to end malnutrition*. https://reliefweb.int/report/world/2020-global-nutrition-report-action-equity-end-malnutrition

[mcn13417-bib-0020] Dewey, K. G. , & Adu‐Afarwuah, S. (2008). Systematic review of the efficacy and effectiveness of complementary feeding interventions in developing countries. Maternal & Child Nutrition, 4(Suppl 1), 24‐85. 10.1111/j.1740-8709.2007.00124.x. 18289157 PMC6860813

[mcn13417-bib-0021] Dewey, K. G. , Hawck, M. G. , Brown, K. H. , Lartey, A. , Cohen, R. J. , & Peerson, J. M. (2005). Infant weight‐for‐length is positively associated with subsequent linear growth across four different populations. Maternal & Child Nutrition, 1(1), 11–20. 10.1111/j.1740-8709.2004.00004.x 16881875 PMC6874388

[mcn13417-bib-0022] Dewey, K. G. , & Mayers, D. R. (2011). Early child growth: How do nutrition and infection interact? Maternal & Child Nutrition, 7(Suppl 3), 129–142. 10.1111/j.1740-8709.2011.00357.x. 21929641 PMC6860756

[mcn13417-bib-0023] DiLalla, L. F. (2008). A structural equation modeling overview for medical researchers. Journal of Developmental and Behavioral Pediatrics, 29(1), 51–54. 10.1097/DBP.0b013e31815f250c 18300720

[mcn13417-bib-0024] Espo, M. , Kulmala, T. , Maleta, K. , Cullinan, T. , Salin, M.‐L. , & Ashorn, P. (2002). Determinants of linear growth and predictors of severe stunting during infancy in rural Malawi. Acta Paediatrica, 91, 1364–1370. 10.1111/j.1651-2227.2002.tb02835.x 12578296

[mcn13417-bib-0025] Fan, Y.‐M. , Oikarinen, S. , Lehto, K.‐M. , Nurminen, N. , Juuti, R. , Mangani, C. , Maleta, K. , Hyöty, H. , & Ashorn, P. (2019). High prevalence of selected viruses and parasites and their predictors in Malawian children. Epidemiology and Infection, 147, e90. 10.1017/S0950268819000025 30869004 PMC6521582

[mcn13417-bib-0026] Hawkes, C. P. , & Grimberg, A. (2015). Insulin‐like growth factor‐I is a marker for the nutritional state. Pediatric Endocrinology Reviews, 13(2), 499–511. 26841638 PMC5576178

[mcn13417-bib-0027] Hoddinott, J. , Behrman, J. R. , Maluccio, J. A. , Melgar, P. , Quisumbing, A. R. , Ramirez‐Zea, M. , Stein, A. D. , Yount, K. M. , & Martorell, R. (2013). Adult consequences of growth failure in early childhood. The American Journal of Clinical Nutrition, 98(5), 1170–1178. 10.3945/ajcn.113.064584 24004889 PMC3798075

[mcn13417-bib-0028] Jackson, B. D. , & Black, R. E. (2017). A literature review of the effect of malaria on stunting. The Journal of Nutrition, 147(11), 2163S–2168S. 10.3945/jn.116.242289 28904111

[mcn13417-bib-0029] Karlberg, J. (1989). A biologically‐oriented mathematical model (ICP) for human growth. Acta Paediatrica Scandinavica, Supplement, 350, 70–94. 10.1111/j.1651-2227.1989.tb11199.x 2801108

[mcn13417-bib-0030] Kosek, M. , Haque, R. , Lima, A. , Babji, S. , Shrestha, S. , Qureshi, S. , Amidou, S. , Mduma, E. , Lee, G. , Yori, P. P. , Guerrant, R. L. , Bhutta, Z. , Mason, C. , Kang, G. , Kabir, M. , Amour, C. , Bessong, P. , Turab, A. , Seidman, J. , … Gottlieb, M. (2013). Fecal markers of intestinal inflammation and permeability associated with the subsequent acquisition of linear growth deficits in infants. The American Journal of Tropical Medicine and Hygiene, 88(2), 390–396. 10.4269/ajtmh.2012.12-0549 23185075 PMC3583335

[mcn13417-bib-0031] Krogvold, L. , Edwin, B. , Buanes, T. , Frisk, G. , Skog, O. , Anagandula, M. , Korsgren, O. , Undlien, D. , Eike, M. C. , Richardson, S. J. , Leete, P. , Morgan, N. G. , Oikarinen, S. , Oikarinen, M. , Laiho, J. E. , Hyöty, H. , Ludvigsson, J. , Hanssen, K. F. , & Dahl‐Jørgensen, K. (2015). Detection of a low‐grade enteroviral infection in the islets of Langerhans of living patients newly diagnosed with type 1 diabetes. Diabetes, 64(5), 1682–1687. 10.2337/db14-1370 25422108

[mcn13417-bib-0032] Lehto, K. M. , Fan, Y. M. , Oikarinen, S. , Nurminen, N. , Hallamaa, L. , Juuti, R. , Mangani, C. , Maleta, K. , Hyöty, H. , & Ashorn, P. (2019). Presence of *Giardia lamblia* in stools of six‐ to 18‐month‐old asymptomatic Malawians is associated with children's growth failure. Acta Paediatrica, 108(10), 1833–1840. 10.1111/apa.14832 31038225 PMC6790611

[mcn13417-bib-0033] Leroy, J. L. , Ruel, M. , Habicht, J.‐P. , & Frongillo, E. A. (2015). Using height‐for‐age differences (HAD) instead of height‐for‐age *z*‐scores (HAZ) for the meaningful measurement of population‐level catch‐up in linear growth in children less than 5 years of age. BMC Pediatrics, 15(1), 145. 10.1186/s12887-015-0458-9 26444012 PMC4595313

[mcn13417-bib-0034] Lufungulo Bahati, Y. , Delanghe, J. , Bisimwa Balaluka, G. , Sadiki Kishabongo, A. , & Philippé, J. (2020). Asymptomatic submicroscopic plasmodium infection is highly prevalent and is associated with anemia in children younger than 5 years in South Kivu/Democratic Republic of Congo. The American Journal of Tropical Medicine and Hygiene, 102(5), 1048–1055. 10.4269/ajtmh.19-0878 32124722 PMC7204578

[mcn13417-bib-0035] Lundeen, E. A. , Stein, A. D. , Adair, L. S. , Behrman, J. R. , Bhargava, S. K. , Dearden, K. A. , Gigante, D. , Norris, S. A. , Richter, L. M. , Fall, C. H. , Martorell, R. , Sachdev, H. S. , & Victora, C. G. , COHORTS Investigators . (2014). Height‐for‐age *z* scores increase despite increasing height deficits among children in 5 developing countries. The American Journal of Clinical Nutrition, 100(3), 821–825. 10.3945/ajcn.114.084368 25008854 PMC4135493

[mcn13417-bib-0036] MAL‐ED Network Investigators . (2017). Childhood stunting in relation to the pre‐ and postnatal environment during the first 2 years of life: The MAL‐ED longitudinal birth cohort study, PLoS medicine, 14(10):e1002408. 10.1371/journal.pmed.1002408 29069076 PMC5656304

[mcn13417-bib-0037] Maleta, K. , Fan, Y.‐M. , Luoma, J. , Ashorn, U. , Bendabenda, J. , Dewey, K. G. , Hyöty, H. , Knip, M. , Kortekangas, E. , Lehto, K.‐M. , Matchado, A. , Nkhoma, M. , Nurminen, N. , Parkkila, S. , Purmonen, S. , Veijola, R. , Oikarinen, S. , & Ashorn, P. (2020). Infections and systemic inflammation are associated with lower plasma concentration of insulin‐like growth factor I among Malawian children. The American Journal of Clinical Nutrition, 113, 380–390. 10.1093/ajcn/nqaa327 PMC7851819 33381802

[mcn13417-bib-0038] Natama, H. M. , Rovira‐Vallbona, E. , Somé, M. A. , Zango, S. H. , Sorgho, H. , Guetens, P. , Coulibaly‐Traoré, M. , Valea, I. , Mens, P. F. , Schallig, H. D. F. H. , Kestens, L. , Tinto, H. , & Rosanas‐Urgell, A. (2018). Malaria incidence and prevalence during the first year of life in nanoro, Burkina Faso: A birth‐cohort study. Malaria Journal, 17(1), 163. 10.1186/s12936-018-2315-4 29650007 PMC5898041

[mcn13417-bib-0039] Nurminen, N. , Juuti, R. , Oikarinen, S. , Fan, Y.‐M. , Lehto, K.‐M. , Mangani, C. , Maleta, K. , Ashorn, P. , & Hyöty, H. (2015). High‐throughput multiplex quantitative polymerase chain reaction method for *Giardia lamblia* and *Cryptosporidium* species detection in stool samples. The American Journal of Tropical Medicine and Hygiene, 92(6), 1222–1226. 10.4269/ajtmh.15-0054 25918202 PMC4458829

[mcn13417-bib-0040] Ozaltin, E. , Hill, K. , & Subramanian, S. V. (2010). Association of maternal stature with offspring mortality, underweight, and stunting in low‐ to middle‐income countries. Journal of the American Medical Association, 303(15), 1507–1516. 10.1001/jama.2010.450 20407060 PMC3100588

[mcn13417-bib-0041] Pickering, A. J. (2019). The WASH benefits and SHINE trials: Interpretation of WASH intervention effects on linear growth and diarrhoea. Health Policy, 7, 8. 10.1016/S2214-109X(19)30268-2 31303300

[mcn13417-bib-0042] Platts‐Mills, J. A. , Babji, S. , Bodhidatta, L. , Gratz, J. , Haque, R. , Havt, A. , McCormick, B. J. , McGrath, M. , Olortegui, M. P. , Samie, A. , Shakoor, S. , Mondal, D. , Lima, I. F. , Hariraju, D. , Rayamajhi, B. B. , Qureshi, S. , Kabir, F. , Yori, P. P. , Mufamadi, B. , … Houpt, E. R. (2015). Pathogen‐specific burdens of community diarrhoea in developing countries (MAL‐ED): A multisite birth cohort study. The Lancet. Global Health, 3(9), e564–e575. 10.1016/S2214-109X(15)00151-5 26202075 PMC7328884

[mcn13417-bib-0043] Prendergast, A. J. , Rukobo, S. , Chasekwa, B. , Mutasa, K. , Ntozini, R. , Mbuya, M. N. N. , Jones, A. , Moulton, L. H. , Stoltzfus, R. J. , & Humphrey, J. H. (2014). Stunting is characterized by chronic inflammation in Zimbabwean infants. PLoS One, 9(2), e86928. 10.1371/journal.pone.0086928 24558364 PMC3928146

[mcn13417-bib-0044] Rogawski, E. T. , Liu, J. , Platts‐Mills, J. A. , Kabir, F. , Lertsethtakarn, P. , Siguas, M. , Khan, S. S. , Praharaj, I. , Murei, A. , Nshama, R. , Mujaga, B. , Havt, A. , Maciel, I. A. , Operario, D. J. , Taniuchi, M. , Gratz, J. , Stroup, S. E. , Roberts, J. H. , Kalam, A. , … Houpt, E. R. , MAL‐ED Network Investigators . (2018). Use of quantitative molecular diagnostic methods to investigate the effect of enteropathogen infections on linear growth in children in low‐resource settings: Longitudinal analysis of results from the MAL‐ED cohort study. The Lancet Global Health, 6(12), e1319–e1328. 10.1016/S2214-109X(18)30351-6 30287125 PMC6227248

[mcn13417-bib-0045] Rosenbloom, A. L. (2007). Physiology of growth. Annales Nestlé, 65(3), 97–108. 10.1159/000112232

[mcn13417-bib-0046] Stewart, C. P. , Iannotti, L. , Dewey, K. G. , Michaelsen, K. F. , & Onyango, A. W. (2013). Contextualising complementary feeding in a broader framework for stunting prevention. Maternal & child nutrition, 9(Suppl 2), 27–45. 10.1111/mcn.12088 24074316 PMC6860787

[mcn13417-bib-0047] Syed, S. , Manji, K. P. , McDonald, C. M. , Kisenge, R. , Aboud, S. , Sudfeld, C. , Locks, L. , Liu, E. , Fawzi, W. W. , & Duggan, C. P. (2018). Biomarkers of systemic inflammation and growth in early infancy are associated with stunting in young Tanzanian children. Nutrients, 10(9), 1158. 10.3390/nu10091158 30149537 PMC6164697

[mcn13417-bib-0048] Thurnham, D. I. , McCabe, L. D. , Haldar, S. , Wieringa, F. T. , Northrop‐Clewes, C. A. , & McCabe, G. P. (2010). Adjusting plasma ferritin concentrations to remove the effects of subclinical inflammation in the assessment of iron deficiency: A meta‐analysis. The American Journal of Clinical Nutrition, 92(3), 546–555. 10.3945/ajcn.2010.29284 20610634

[mcn13417-bib-0049] United Nations Children's Fund (UNICEF), World Health Organization & The World Bank . (2020). *Levels and trends in child malnutrition: Key findings of the 2020 edition of the joint child malnutrition estimates*. World Health Organization.

[mcn13417-bib-0050] Victora, C. G. , de Onis, M. , Hallal, P. C. , Blössner, M. , & Shrimpton, R. (2010). Worldwide timing of growth faltering: Revisiting implications for interventions. Pediatrics, 125(3), e473–e480. 10.1542/peds.2009-1519 20156903

[mcn13417-bib-0051] Vu, D. T. , Sethabutr, O. , Von Seidlein, L. , Tran, V. T. , Do, G. C. , Bui, T. C. , Le, H. T. , Lee, H. , Houng, H.‐S. , Hale, T. L. , Clemens, J. D. , Mason, C. , & Dang, D. T. (2004). Detection of Shigella by a PCR assay targeting the ipaH gene suggests increased prevalence of shigellosis in Nha Trang, Vietnam. Journal of Clinical Microbiology, 42(5), 2031–2035. 10.1128/jcm.42.5.2031-2035.2004 15131166 PMC404673

[mcn13417-bib-0052] Walters, T. D. , & Griffiths, A. M. (2009). Mechanisms of growth impairment in pediatric Crohn's disease. Nature Reviews Gastroenterology & Hepatology, 6(9), 513–523. 10.1038/nrgastro.2009.124 19713986

[mcn13417-bib-0053] Weisz, A. , Meuli, G. , Thakwalakwa, C. , Trehan, I. , Maleta, K. , & Manary, M. (2011). The duration of diarrhea and fever is associated with growth faltering in rural Malawian children aged 6‐18 months. Nutrition Journal, 10(1), 25. 10.1186/1475-2891-10-25 21418600 PMC3068082

[mcn13417-bib-0057] World Health Organization . (2006). WHO child growth standards: Length/height‐forage, weight‐for‐age, weight‐for‐length, weight‐for‐height and body mass index‐for‐age: Methods and development. World Health Organization.

[mcn13417-bib-0054] World Health Organization . (2012). *Maternal, infant and young child nutrition.* Sixty‐fifth World Health Assembly, Resolutions and Decision (Resolution WHA65.6.). http://apps.who.int/gb/ebwha/pdf_files/WHA65/A65_11-en.pdf

[mcn13417-bib-0055] Wong, S. C. , Dobie, R. , Altowati, M. A. , Werther, G. A. , Farquharson, C. , & Ahmed, S. F. (2016). Growth and the growth Hormone‐Insulin like growth factor 1 axis in children with chronic inflammation: Current evidence, gaps in knowledge, and future directions. Endocrine Reviews, 37(1), 62–110. 10.1210/er.2015-1026 26720129

