# Supplementary material for: Association between asymptomatic infections and linear growth in 18–24‐month‐old Malawian children
Source: Matern Child Nutr. 2022 Sep 16;19(1):e13417. doi: 10.1111/mcn.13417 (PMC9749610; doi:10.1111/mcn.13417)
Supplement: Supplementary file 1 — Supporting information. [file MCN-19-e13417-s001.doc]

# **Supplementary Figures and Supplementary Tables**

**Legends of Supplementary Figures:**

**Supplementary Figure 1.** Conceptual map of the hypothesized direct and indirect associations with LAZ at 24 months. Lines in blue are hypothesized positive associations and lines in red represent negative hypothesized associations. Dashed lines from shaded boxes represent factors that we believe to exist but that we couldn’t test due to lack of data in our data set. A1AT, alpha-1-antitrypsin; AGP, alpha-1-acid glycoprotein; CXM, collagen X biomarker; IGF-1, insulin-like growth factor 1; LAZ, length-for-age Z score; WLZ, weight-for-length Z score.

**Supplementary Figure 2.** Flow diagram of participants.

**Supplementary Table 1.** Baseline characteristics of participants.

**Supplementary Table 2.** Missing data for each variable among 604 study participants.

**Supplementary Table 3.** The Association Between Socio-Economic, Maternal or Child Factors and Children’s LAZ at 24 Mo

**Supplementary Table 4.** Coefficients between variables using the structural equation model (SEM)

**Supplementary Figure 1.**


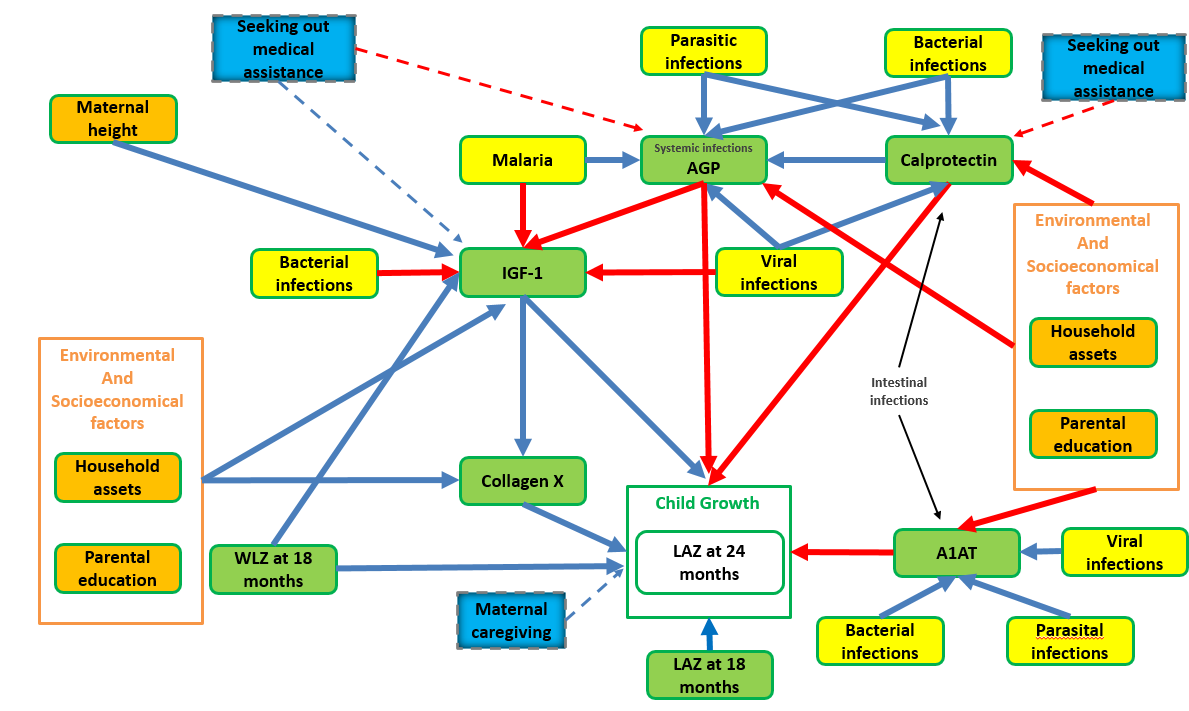


**Supplementary Figure 2.**


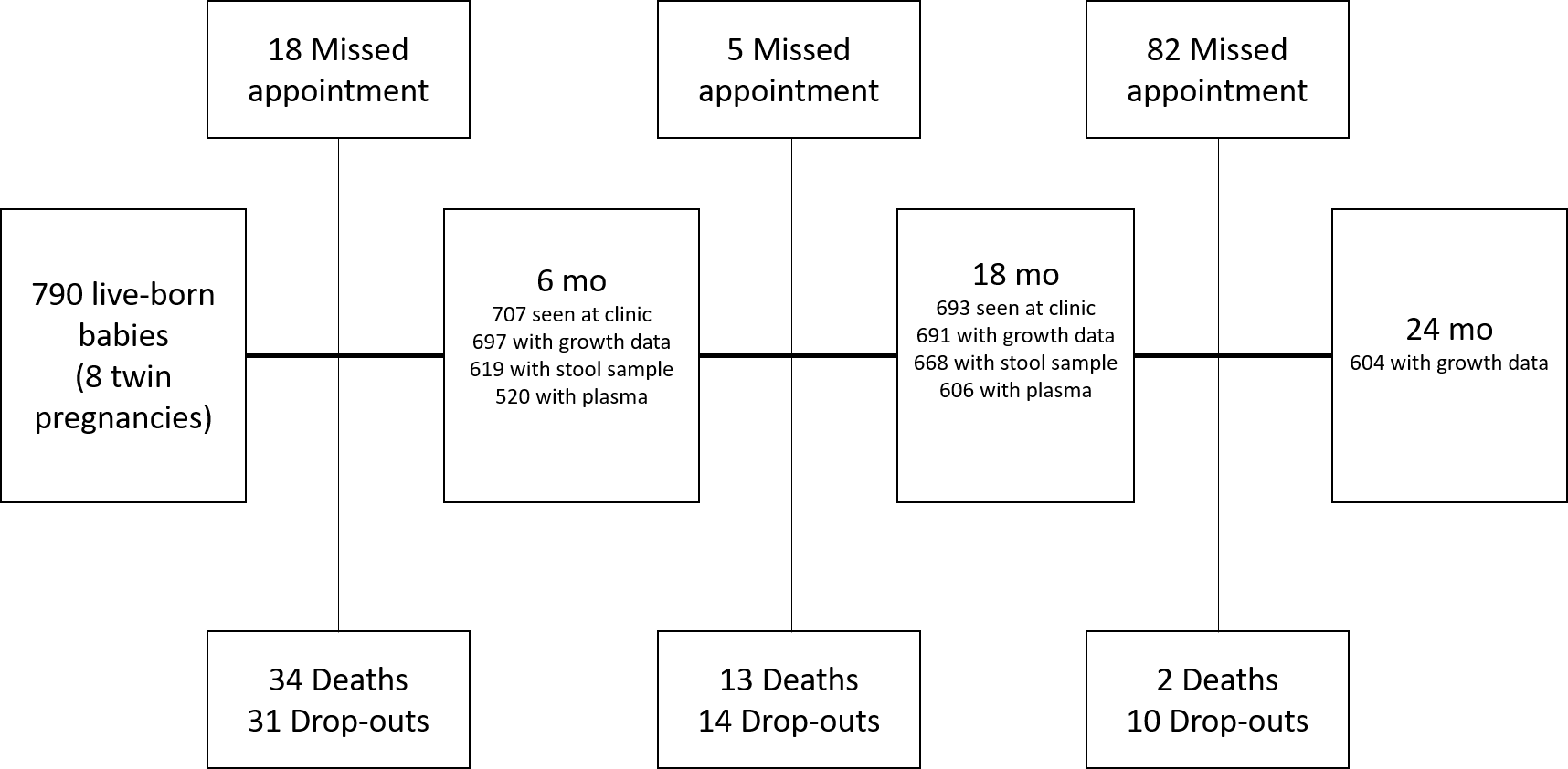


**Supplementary Table 1. Baseline characteristics of study participants**

| **Characteristic** | **Included in analysis** | **Excluded from analysis** | ***P* value^a^** |
| --- | --- | --- | --- |
| Number of participants, *n* | 604 | 186 |  |
| Proportion of boys, *%* | 48.2 | 46.0 | 0.614 |
| Maternal height, cm | 156.1 ± 5.7 | 156.3 ± 5.9 | 0.691 |
| Primiparous, *%* | 17.8 | 36.0 | <0.001 |
| Maternal HIV positive status, *%* | 12.5 | 10.7 | 0.521 |
| Maternal education, *y* | 3.6 ± 3.4 | 4.8 ± 3.8 | <0.001 |
| Unimproved sanitation facilities,^b^ *%* | 91.5 | 86.8 | 0.057 |
| Unsafe water source,^c^ % | 8.5 | 9.9 | 0.536 |

Values are means ± SDs or percentages unless otherwise indicated.

^a^*P* value obtained using Student’s t-test for continuous variables and χ^2^ test for categorical variables.

^b^Regular pit latrine or no latrine.

^c^Unprotected well, lake, or pond.

**Supplementary Table 2. Missing data for each variable among 604 study participants**

| **Variable** | **No. of missing data** | **Percentage (%)** |
| --- | --- | --- |
| Length-for-age Z score at 24 mo | 0 | 0 |
| Length-for-age Z score at 18 mo | 3 | 0.5 |
| Weight-for-length Z score at 18 mo | 3 | 0.5 |
| Calprotectin at 18 mo | 18 | 3.0 |
| Alpha 1 antitrypsin at 18 mo | 43 | 7.1 |
| *Campylobacter* at 18 mo | 15 | 2.5 |
| Shigella at 18 mo | 17 | 2.8 |
| *Cryptosporidium* at 18 mo | 18 | 3.0 |
| *Giardia* *lamblia* at 18 mo | 18 | 3.0 |
| Enterovirus at 18 mo | 18 | 3.0 |
| Parechovirus at 18 mo | 18 | 3.0 |
| Norovirus at 18 mo | 18 | 3.0 |
| Rhinovirus at 18 mo | 18 | 3.0 |
| Rotavirus at 18 mo | 18 | 3.0 |
| Malaria at 18 mo | 25 | 4.1 |
| Alpha-1-acid glycoprotein at 18 mo | 55 | 9.1 |
| Insulin-like growth factor 1 at 18 mo | 64 | 10.6 |
| Collagen X biomarker at 18 mo | 91 | 15.1 |
| Newborn child sex | 0 | 0 |
| Maternal education | 4 | 0.7 |
| Maternal height | 3 | 0.5 |
| Household asset Z score | 1 | 0.2 |

**Supplementary Table 3. The Association Between Socio-Economic, Maternal or Child Factors and Children’s LAZ at 24 Mo**

|  |  | Mean LAZ at 24 mo, by the participants’ rank in the indicated predictor variable value distribution | | | | | |
| --- | --- | --- | --- | --- | --- | --- | --- |
| Predictor variable | Mean ± SD predictor value | Lowest quintile ^a^ | 2^nd^ quintile | 3^rd^ quintile | 4^th^ quintile | Highest quintile | *P* value ^b^ |
| LAZ at 18 mo | -1.7 ± 1.1 | -2.99 | -2.26 | -1.79 | -1.29 | -0.52 | <0.001 |
| WLZ at 18 mo | -0.2 ± 1.0 | -2.44 | -1.83 | -1.74 | -1.42 | -1.44 | <0.001 |
| Maternal height | 156.1 ± 5.7 cm | -2.24 | -2.06 | -1.82 | -1.52 | -1.22 | <0.001 |
| Maternal education | 3.6 ± 3.4 y | -1.85 | -1.64 | -1.80 | -1.90 | -1.54 | 0.20 |
| Household asset Z-score | -0.1 ± 0.9 | -1.89 | -1.52 | -1.71 | -1.63 | -1.52 | 0.002 |

^a^ Children within the lowest quintile of the predictor variable (lowest 20% of values).

^b^ *P* value obtained using Cuzick’s Wilcoxon-type test for trend.

Abbreviations: LAZ, length-for-age Z score; WLZ, weight-for-length Z score.

**Supplementary Table 4. Coefficients between variables using the structural equation model (SEM)**

| **Response variable** | **Predictors** | **Coefficient (SE)** | ***P* value** |
| --- | --- | --- | --- |
| LAZ at 24 mo | LAZ at 18 mo | 0.79 (0.02) | <0.001 |
|  | WLZ at 18 mo | 0.08 (0.02) | <0.001 |
|  | IGF-1 at 18 mo | 0.07 (0.02) | 0.001 |
|  | CXM at 18 mo | 0.05 (0.02) | 0.018 |
|  | Maternal height | 0.04 (0.02) | 0.062 |
|  | Shigella at 18 mo | -0.19 (0.07) | 0.008 |
| IGF-1 at 18 mo | WLZ at 18 mo | 0.13 (0.04) | 0.001 |
|  | AGP at 18 mo | -0.29 (0.04) | <0.001 |
|  | Enterovirus at 18 mo | -0.26 (0.11) | 0.014 |
|  | *Shigella* at 18 mo | -0.25 (0.14) | 0.077 |
|  | Newborn child sex (girl) | 0.32 (0.08) | <0.001 |
|  | Maternal height | 0.13 (0.04) | 0.002 |
| CXM at 18 mo | IGF-1 at 18 mo | 0.13 (0.04) | 0.003 |
|  | AGP at 18 mo | -0.13 (0.05) | 0.007 |
|  | Malaria at 18 mo | -0.39 (0.14) | 0.007 |
|  | Newborn child sex (girl) | 0.24 (0.09) | 0.004 |
|  | Enterovirus at 18 mo | 0.19 (0.12) | 0.103 |
| AGP at 18 mo | Malaria at 18 mo | 0.87 (0.13) | <0.001 |
|  | *Shigella* at 18 mo | 0.37 (0.14) | 0.009 |
|  | *Campylobacter* at 18 mo | 0.20 (0.09) | 0.027 |
|  | Calprotectin at 18 mo | 0.10 (0.04) | 0.012 |
|  | Norovirus at 18 mo | 0.28 (0.16) | 0.074 |
| Calprotectin at 18 mo | *Shigella* at 18 mo | 0.29 (0.14) | 0.048 |
|  | *Campylobacter* at 18 mo | 0.19 (0.09) | 0.036 |
|  | Rhinovirus at 18 mo | 0.58 (0.19) | 0.003 |
|  | Norovirus at 18 mo | 0.30 (0.15) | 0.052 |

Abbreviations: AGP, alpha-1-acid glycoprotein; CXM, collagen X biomarker; IGF-1, insulin-like growth factor; LAZ, length-for-age Z score; WLZ, weight-for-length Z score; SE, Standard Error
